# A Novel Necroptosis-Related Gene Signature in Skin Cutaneous Melanoma Prognosis and Tumor Microenvironment

**DOI:** 10.3389/fgene.2022.917007

**Published:** 2022-07-11

**Authors:** Binyu Song, Pingfan Wu, Zhen Liang, Jianzhang Wang, Yu Zheng, Yuanyong Wang, Hao Chi, Zichao Li, Yajuan Song, Xisheng Yin, Zhou Yu, Baoqiang Song

**Affiliations:** ^1^ Department of Plastic Surgery, Xijing Hospital, Fourth Military Medical University, Xi'an, China; ^2^ Department of Burn and Plastic Surgery, The First Affiliated Hospital of Soochow University, Suzhou, China; ^3^ Hospital for Skin Disease (Institute of Dermatology), Chinese Academy of Medical Sciences and Peking Union Medical College, Nanjing, China; ^4^ Department of Thoracic Surgery, Tangdu Hospital of Air Force Military Medical University, Xi'an, China; ^5^ Clinical Medical College, Southwest Medical University, Luzhou, China

**Keywords:** necroptosis, prognostic signature, SKCM, TCGA, TME

## Abstract

**Background:** Necroptosis has been identified recently as a newly recognized programmed cell death that has an impact on tumor progression and prognosis, although the necroptosis-related gene (NRGs) potential prognostic value in skin cutaneous melanoma (SKCM) has not been identified. The aim of this study was to construct a prognostic model of SKCM through NRGs in order to help SKCM patients obtain precise clinical treatment strategies.

**Methods:** RNA sequencing data collected from The Cancer Genome Atlas (TCGA) were used to identify differentially expressed and prognostic NRGs in SKCM. Depending on 10 NRGs *via* the univariate Cox regression analysis usage and LASSO algorithm, the prognostic risk model had been built. It was further validated by the Gene Expression Omnibus (GEO) database. The prognostic model performance had been assessed using receiver operating characteristic (ROC) curves. We evaluated the predictive power of the prognostic model for tumor microenvironment (TME) and immunotherapy response.

**Results:** We constructed a prognostic model based on 10 NRGs (FASLG, TLR3, ZBP1, TNFRSF1B, USP22, PLK1, GATA3, EGFR, TARDBP, and TNFRSF21) and classified patients into two high- and low-risk groups based on risk scores. The risk score was considered a predictive factor in the two risk groups regarding the Cox regression analysis. A predictive nomogram had been built for providing a more beneficial prognostic indicator for the clinic. Functional enrichment analysis showed significant enrichment of immune-related signaling pathways, a higher degree of immune cell infiltration in the low-risk group than in the high-risk group, a negative correlation between risk scores and most immune checkpoint inhibitors (ICIs), anticancer immunity steps, and a more sensitive response to immunotherapy in the low-risk group.

**Conclusions:** This risk score signature could be applied to assess the prognosis and classify low- and high-risk SKCM patients and help make the immunotherapeutic strategy decision.

## Introduction

Skin cutaneous melanoma (SKCM) is considered aggressive cancer. Its global prevalence is 15–25 individuals per 100,000 with an annual increase of 3–5% (Schadendorf et al., 2015). Early-stage melanoma with timely surgery showed a favorable prognosis. The 10-year survival rate was up to 95%, while the rate in metastatic melanoma was less than 20% (Balch et al., 2009; Gershenwald et al., 2017). The treatment of advanced melanoma is limited and mainly depends on immunotherapy (Leonardi et al., 2020). Overall, SKCM patients remain at a high recurrence rate with all kinds of interventions. So, identifying effective prognostic biomarkers is a must to develop better prognosis methods.

Apoptosis and necroptosis are both programmed cell death mechanisms. One is natural (Bertheloot et al., 2021), and the other is caspase-independent. Necroptosis is used to treat tumors after drug resistance to apoptosis, and is mediated by the toll-like and tumor necrosis factor (TNF) receptor activation. The receptor-interacting protein kinase 1, 3 (RIPK1) (RIPK3), and their target—the mixed lineage kinase domain-like protein (MLKL)—are three key proteins to initiate necroptosis (Gong et al., 2019; Bertheloot et al., 2021). Necroptosis plays a tumor-inhibiting role in most cases (Tang et al., 2020). Previous studies found that necroptosis-related regulatory factors could be a biomarker for the prognosis of tumors and some diseases (Zhang et al., 2018; Park et al., 2020). For instance, in glioblastoma, Park et al. indicated that the overexpression of RIPK1 is correlated with a poorer prognosis (Park et al., 2009). Low RIPK3 expression and poor prognosis are correlated (Feng et al., 2015). The potential role of necroptosis in tumors has stimulated intense research interest. However, the role of necroptosis in SKCM is rarely reported.

This study aims to elucidate the NRGs expression and prognostic significance in SKCM. To assess the NRGs prognostic value in SKCM, we established a survival-based risk score model. The study findings may provide clues for prognostic biomarkers in SKCM and focus on individual-specific SKCM treatment.

## Materials and Methods

### Data Collection

The TCGA had been used to collect the SKCM patients’ clinical data and mRNA expression. The Genotype-Tissue Expression (GTEx) database had been used to collect the transcriptome data of 556 normal skin samples. To assemble an internal training group, the TCGA-SKCM (*n* = 471) patients were recruited. The GSE54467 and GSE65904 datasets collected from the Gene Expression Omnibus (GEO) had been used as a validation set (*n* = 293), which is used for external validation of the model. The R “Limma” package had been used to process and merge data collected from GTEx and TCGA (Law et al., 2016). The microarray data GSE54467 and GSE65904 had been also merged and standardized using the R package “Limma” usage.

### Differentially Expressed Gene Identification

Sixty-seven NRGs had been collected from previously published studies and the Gene Set Enrichment Analysis (GSEA) (Supplementary Table S1). A differential gene expression analysis with a |log2FC| > 1 and FDR < 0.05 had been done between tumor and normal tissues using the “limma” R package. The relationship’s significance between overall survival (OS) and all NRGs in TCGA-SKCM was assessed using the univariate Cox regression analysis with a *p* <0.05 cutoff, which was done by the “survival” R package usage. The Venn diagram package was used to produce overlapping results of DEGs and prognostic genes as a graphical output and candidate NRGs. Interaction networks for the 32 prognostic NRGs and overlapping prognostic DEGs were analyzed using the R packages “igraph” and “psych”.

### Establishment and Validation for the Necroptosis-Related Genes Prognostic Signature

To avoid the overfitting risk, we incorporated the candidate NRGs into LASSO-penalized Cox regression analysis using the R package “glmnet” to select hub genes and build a gene risk signature (Tibshirani, 1997; Simon et al., 2011). This formula *Risk score = sum (each gene’s expression × corresponding coefficient)* was used for calculating the risk score. Considering the medium risk score, the SKCM patients were categorized into two risk groups. Kaplan–Meier survival analysis had been done using the R package “survival” and “survminer” for evaluating the two groups’ OS. The validation set, including GSE54467 and GSE65904 merged, was used for the external evaluation. To perform 2-, 3-, 5-, 7-, and 10-year receiver operating characteristic (ROC) analyses, the R package “timeROC” was used. The prognostic model by univariate independent prognostic analysis and multivariate independent prognostic analysis using the R package “survival” was built for identifying the clinical features, risk score, and patient OS correlation. Using the R package “rms,” a nomogram was constructed. To assess the nomogram’s prognostic accuracy, calibration and ROC curves were performed.

### Functional Enrichment Analyses

The DEGs (|log2FC|≥ 1 and FDR < 0.05) were filtered in TCGA-SKCM among the two risk groups. The Gene Ontology (GO) functional enrichment analysis had been done for the DEGs using the R “clusterProfiler” package and “circlize” package. The Kyoto Encyclopedia of Genes and Genomes (KEGG) enrichment analysis associated with the NRG signature had been done by conducting the Gene Set Variation Analysis (GSVA) (Hänzelmann et al., 2013; Kanehisa et al., 2021). The R package “GSVA” was used to find enriched pathways between the two risk groups using a normalized *p* < 0.05.

### Immune Response and Tumor Microenvironment Analysis

The EPIC (Racle and Gfeller, 2020), MCP-counter (Becht et al., 2016), XCELL (Aran et al., 2017), QUANTISEQ (Finotello et al., 2019), CIBERSORT-ABS, CIBERSORT (Newman et al., 2015; Chen et al., 2018), and TIMER (Li et al., 2020) algorithms had been used for calculating the relationship between the risk score and immune filtration status. For calculating the immune score that determines the immune-stromal component levels of the tumor samples’ ratio by the ESTIMATE algorithm, the R package “estimate” was used. These scores were Immune, Stromal, and ESTIMATE scores. Each of them was linked to immune and stromal cells and their sum in TME. We evaluated the correlation between risk scores and tumor stemness, and the relationship between immune infiltration subtypes and risk scores using the two-way Spearman correlation. The cancer immunity cycle gene set was derived from tracking tumor immunophenotype (TIP; http://biocc.hrbmu.edu.cn/TIP/), and the ssGSEA algorithm enriched the cancer-immune cycle–related gene set between the two risk groups and analyzed the correlation between risk score and cancer-immune cycle.

### Immunotherapy Analysis

To assess the response to the immunotherapy in risk subgroups, we used the Cancer Immunome Database (TCIA) to obtain the SKCM patients’ Immunophenoscores (IPS) and then compared the differences in IPS between risk groups.

### Online Database Verification

The HPA database was used to identify ten NRGs’ protein expression levels in tumor and normal tissues (Uhlen et al., 2010). The K-M survival curves showed prognostic significances of NRGs, in which the patients were separated based on each gene’s median expression into two groups by the TIMER 2.0 usage (Li et al., 2017).

### Tumor Immune Single-Cell Hub Database

The Tumor Immune Single-Cell Hub (TISCH; http://tisch.comp-genomics.org) is a large-scale online database of single-cell RNA-seq focused on the TME (Sun et al., 2021). This database was used to systematically investigate the TME heterogeneity in various datasets and cell types.

## Results

### Prognostic Necroptosis-Related DEGs Identification in The Cancer Genome Atlas Cohort

**FIGURE 1:**
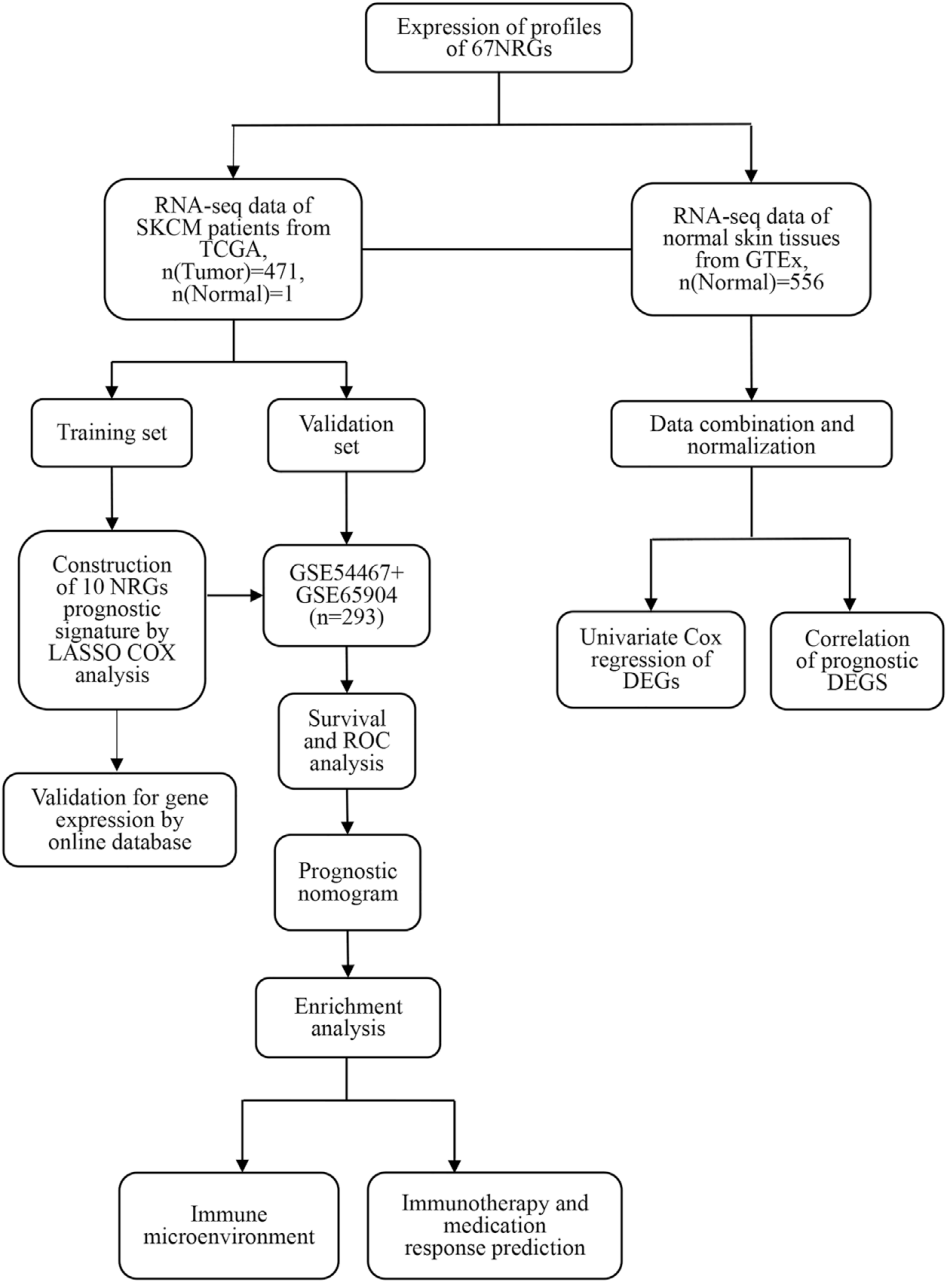
The study workflow.

**FIGURE 2:**
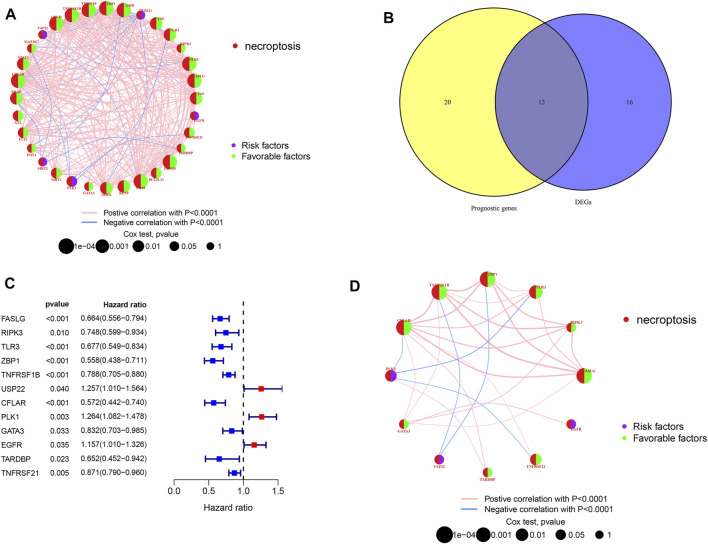
Candidate prognostic DEGs identification in TCGA-SKCM. **(A,D)** Interactions between prognostic NRGs and prognostic DEGs in SKCM. The lines connecting the NRGs represent the correlations, and thicker lines represent larger correlations. Pink and blue represent positive and negative correlations. **(B)** Venn diagram of NRGs identified by univariate Cox analyses and differential expression. **(C)** Forest plots of correlations between 12 NRGs and OS of patients in TCGA cohort.

Figure 1 shows the study design workflow diagram and grouping. Thirty-two prognostic genes were chosen from 67 NRGs using univariate Cox regression, and their network was presented (Figure 2A). Sixty-seven NRGs’ expression levels were examined in 557 normal skins and 471 melanoma tissues from TCGA and GTEx datasets, and 28 genes were differentially expressed. FASLG, RIPK3, TLR3, ZBP1, TNFRSF1B, USP22, CFLAR, PLK1, GATA3, EGFR TARDBP, and TNFRSF21 were significantly related to the patient’s OS. These genes were considered the prognostic necroptosis-related DEGs (Figure 2B). A univariate Cox regression analysis was done to determine 12 candidate genes (Figure 2C). The correlation analysis of these genes was performed in Figure 2D. The heatmap revealed the 12 candidate genes’ differential expression in tumor tissues and normal skin (Supplementary Figure S1).

### Gene Signature Construction in The Cancer Genome Atlas and Gene Expression Omnibus Cohort

**FIGURE 3:**
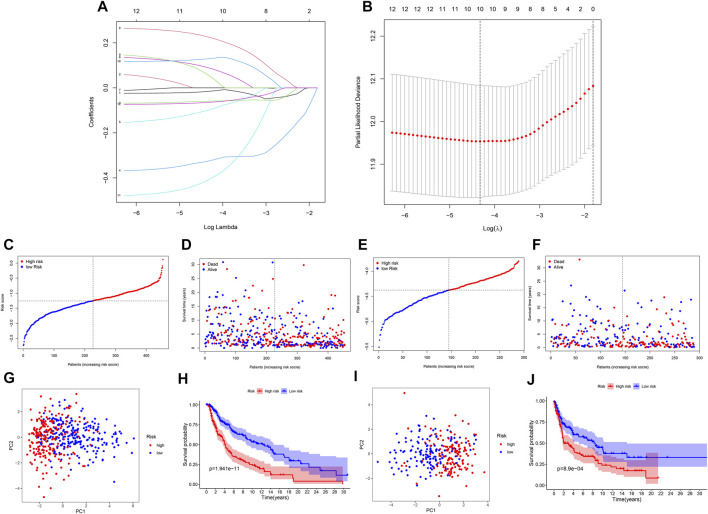
A prognostic signature of NRG construction. **(A)** The 10 prognostic genes’ LASSO coefficient profiles. **(B)** Partial likelihood deviation map. **(C,F)** Patients in TCGA and GEO cohorts’ risk score distribution and survival status. **(G,I)** TCGA and GEO cohorts’ PCA plots. **(H,J)** K-M survival curves for the training and validation sets.

**FIGURE 4:**
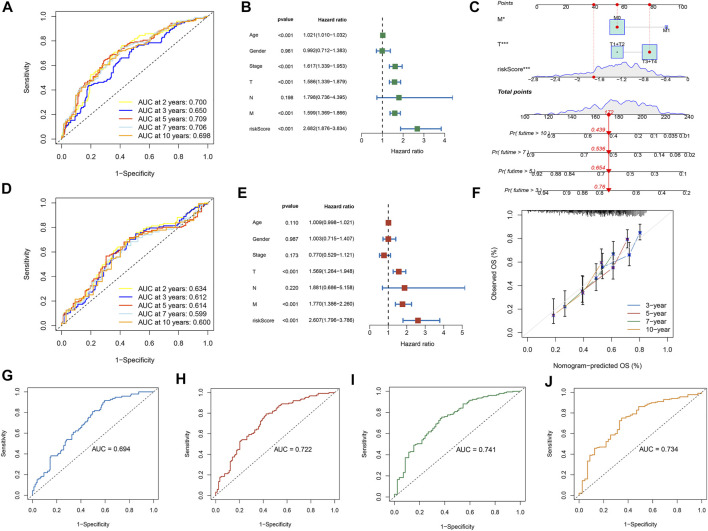
The risk model performance. **(A,D)** The prognostic model ROC curves in TCGA and GEO cohorts. The univariate **(B)** and multivariate **(E)** Cox regression analysis of factors with OS. Construction of the nomogram model: **(C)** Nomogram predicting 3-, 5-, 7- and 10-year OS for SKCM patients. **(F)** Nomogram model calibration curves. **(G–J)** The nomogram’s time-dependent ROC curves predict 3-, 5-, 7-, and 10-year survival.

**FIGURE 5:**
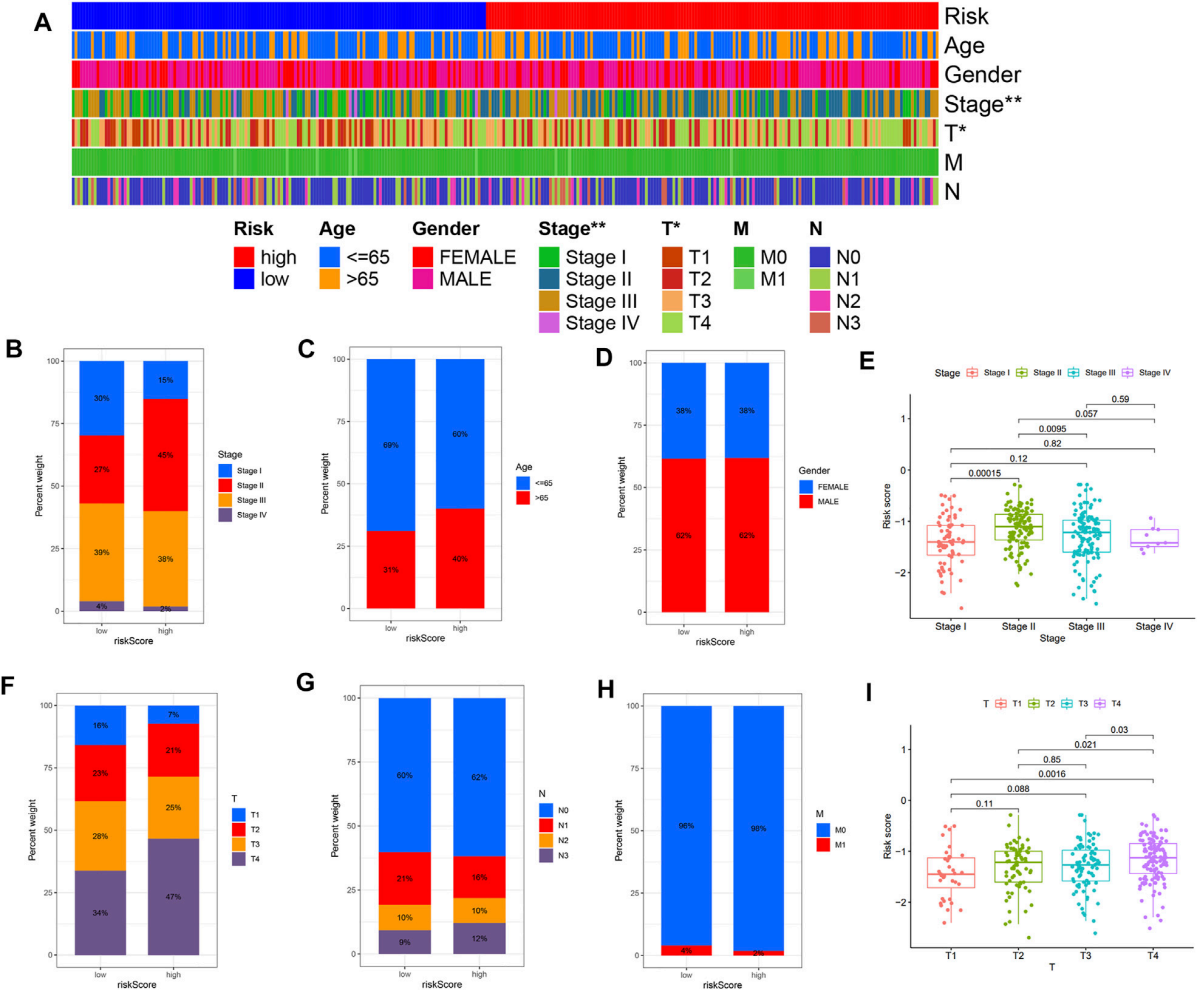
The risk score and clinicopathological characteristics’ correlation. **(A)** The heatmap shows the clinicopathological features distribution between the two risk subgroups. **(B)** Risk score distribution stratified by **(B)** tumor stage, **(C)** age, **(D)** gender, **(F)** T stage, **(G)** N stage, and **(H)** M stage. **(E,I)** The distribution of risk scores according to tumor stage and T stage. *p <0.05, **p <0.01 and ***p <0.001.

Further LASSO analysis was done to construct a prognostic signature with 12 prognostic DEGs, we used data from TCGA as a training set, and finally, we selected ten genes from 12 prognostic DEGs (Table 1). To determine the penalty parameter (λ), the minimum parameters had been used (Figures 3A,B). GEO data were used as a validation set for the external evaluation. Patients in these two cohorts were categorized into two risk subgroups based on the median risk scores (Figures 3C–F). The PCA analysis findings suggested that the two subgroups’ patients were distributed randomly through the TCGA (Figure 3G) and GEO (Figure 3I). The K-M curve suggested that the risk levels could significantly predict the OS in SKCM patients (Figures 3H,J). The OS of the low-risk subgroup increased in the two cohorts. The risk model’s predictive accuracy is moderate according to the ROC curves at years 2 (ROC = 0.700), 3 (ROC = 0.650), 5 (ROC = 0.709), 7 (ROC = 0.706), and 10 (ROC = 0.698) (Figure 4A). Furthermore, the results in the validation set were also obtained (Figure 4D). The risk score could function as a predictive factor for patients in the TCGA cohort. Both univariate and multivariate Cox regression analyses were used for analyzing age, gender, tumor stage, TNM stage, and risk score. The risk score and OS were linked in the univariate analysis (HR = 2.682, 95%CI = 1.876–3.834, *p* < 0.001) (Figure 4B). In the multivariate Cox regression analysis, they were shown to be an independent OS predictor (HR = 2.607, 95% CI = 1.796–3.786, *p* < 0.001) (Figure 4E). These findings indicated that the risk score was a predictive factor. Based on the TCGA cohort, for 471 SKCM patients, a nomogram was employed to predict the 3-, 5-, 7-, and 10-year OS (Figure 4C). Figure 4F presented the nomogram’s high accuracy and sensitivity in a calibration plot. The 3-, 5-, 7-, and 10-year AUC values were 0.694, 0.722, 0.741, and 0.734, respectively, in the training cohort (Figures 4G–J). By comparing the distribution of clinical features of the high- and low-risk groups, we observed a significant difference in the distribution of the tumor stage and T stage in the risk group, while other clinical characteristics did not change significantly between the two subgroups (Figures 5A–D, F−H). Furthermore, we performed a comprehensive analysis of the distribution of risk scores in tumor stage and T stage, and we found that patients with high T-stage tumors had higher risk scores (Figures 5E,I).

**TABLE 1:** Coefficients in the LASSO Cox regression model.

i	Gene	Coef
1	FASLG	−0.01553
2	TLR3	−0.059485
3	ZBP1	−0.324751
4	TNFRSF1B	−0.098664
5	USP22	0.088670
6	PLK1	0.225349
7	GATA3	0.055237
8	EGFR	0.129443
9	TARDBP	−0.408012
10	TNFRSF21	−0.064455

### Functional Enrichment Analysis

**FIGURE 6:**
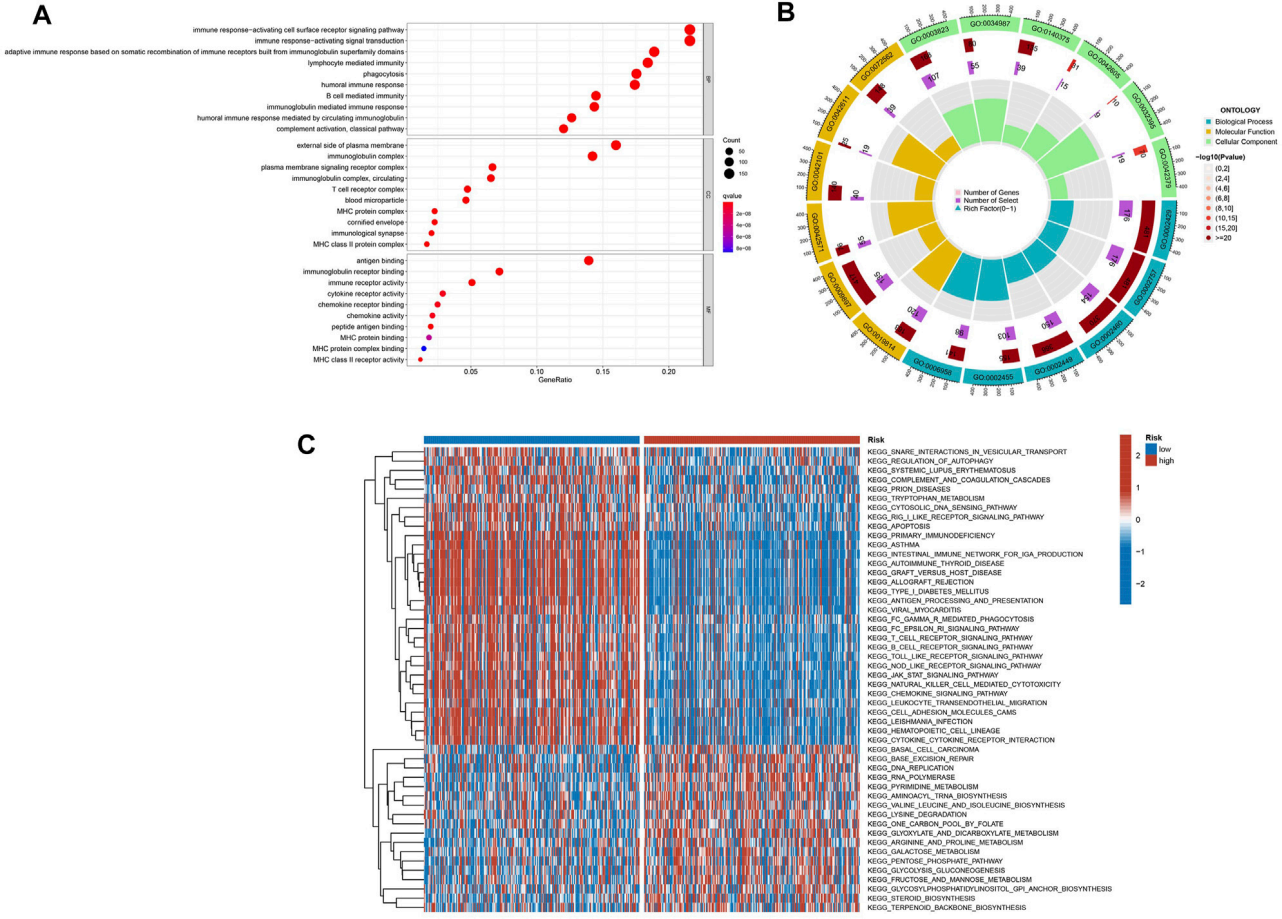
The enriched items in functional analysis. **(A,B)** Gene ontology (GO) enrichment analysis. **(C)** KEGG pathway enrichment by GSVA between two risk subgroups.

GO analyses were performed to assess the two risk groups’ DEGs for clarifying the biological activities and the risk score’s correlation. Biological process (BP), cellular component (CC), and molecular function (MF) were the GO enrichment analyses’ three parts. For the TCGA database, the enriched GO terms of each part were shown in Figures 6A,B and Supplementary Table S2. The major biological process was connected with the immunity-related process, such as immune response–activating cell surface receptor signaling pathway, leukocyte-mediated immunity, immune response–regulating signaling pathway, and immune response–activating signal transduction. The GSVA was performed to analyze KEGG pathways between two risk subgroups, revealing 57 significantly enriched pathways (adj *p*-value <0.05; Supplementary Table S3). The GSVA-KEGG pathway enrichment in the low-risk group was significantly related to immunity, including the B-cell receptor signaling pathway, toll-like receptor signaling, T-cell receptor signaling pathway, and natural killer cell–mediated cytotoxicity (Figure 6C). We were surprised to find that many GO terms and KEGG pathways were linked to immunological responses. Therefore, we further investigated the correlation between immune response and the risk score.

### Immune Response and Tumor Microenvironment

**FIGURE 7:**
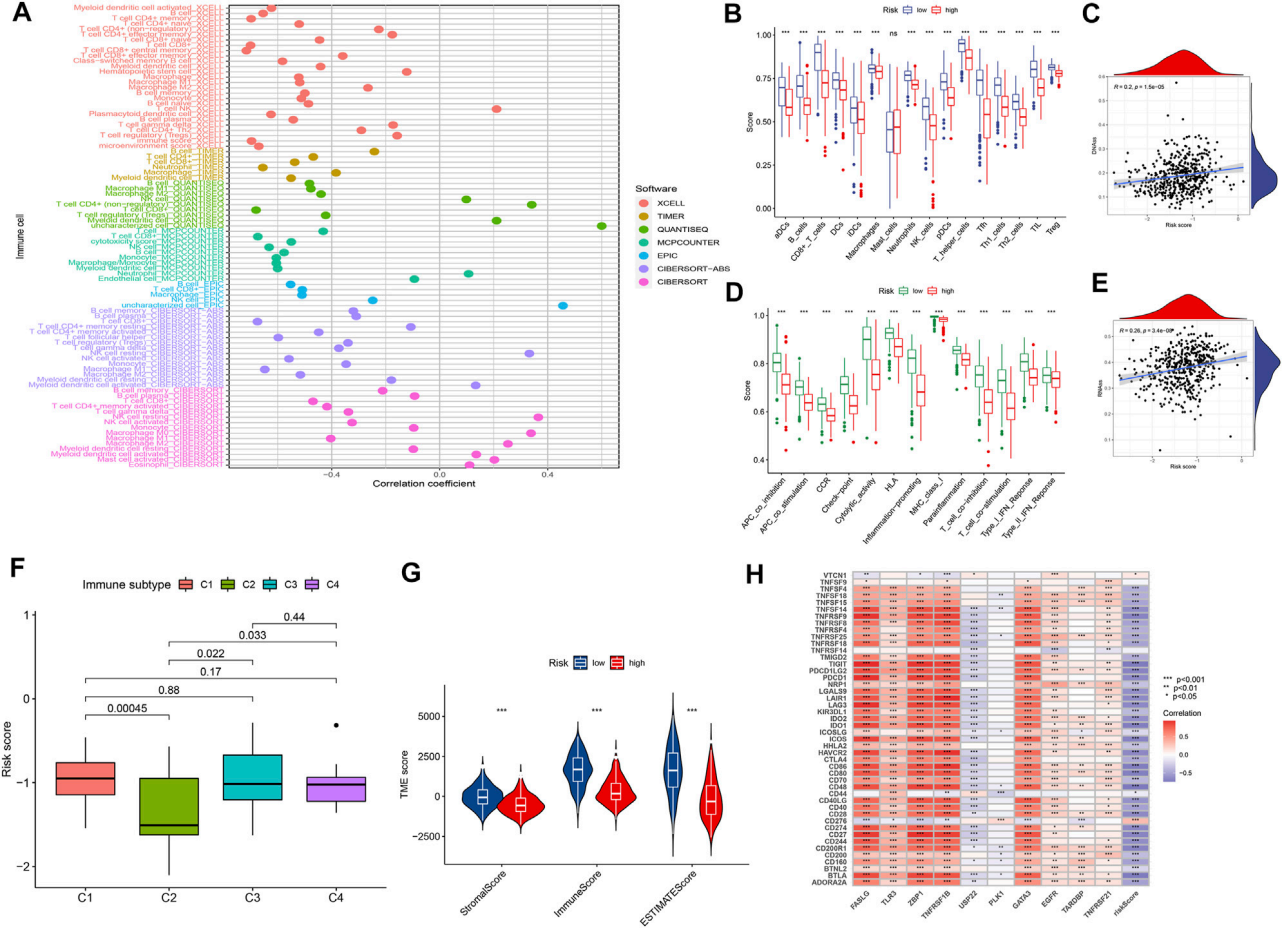
The tumor microenvironment characteristics between risk subgroups. **(A)** Immune cell infiltration based on XCELL, TIMER, QUANTISEQ, MCP-counter, CIBERSORT, CIBERSORT-ABS, and EPIC algorithms between high- and low-risk groups. **(B,D)** Comparison of ssGSEA scores between the two risk groups in the TCGA cohort. **(G)** ESTIMATE scores, stromal scores, and immune scores for different risk statuses. **(C,E,F,H)** Correlation between risk scores and RNAs, DNAs, immune subtype, and immune checkpoints.

**FIGURE 8:**
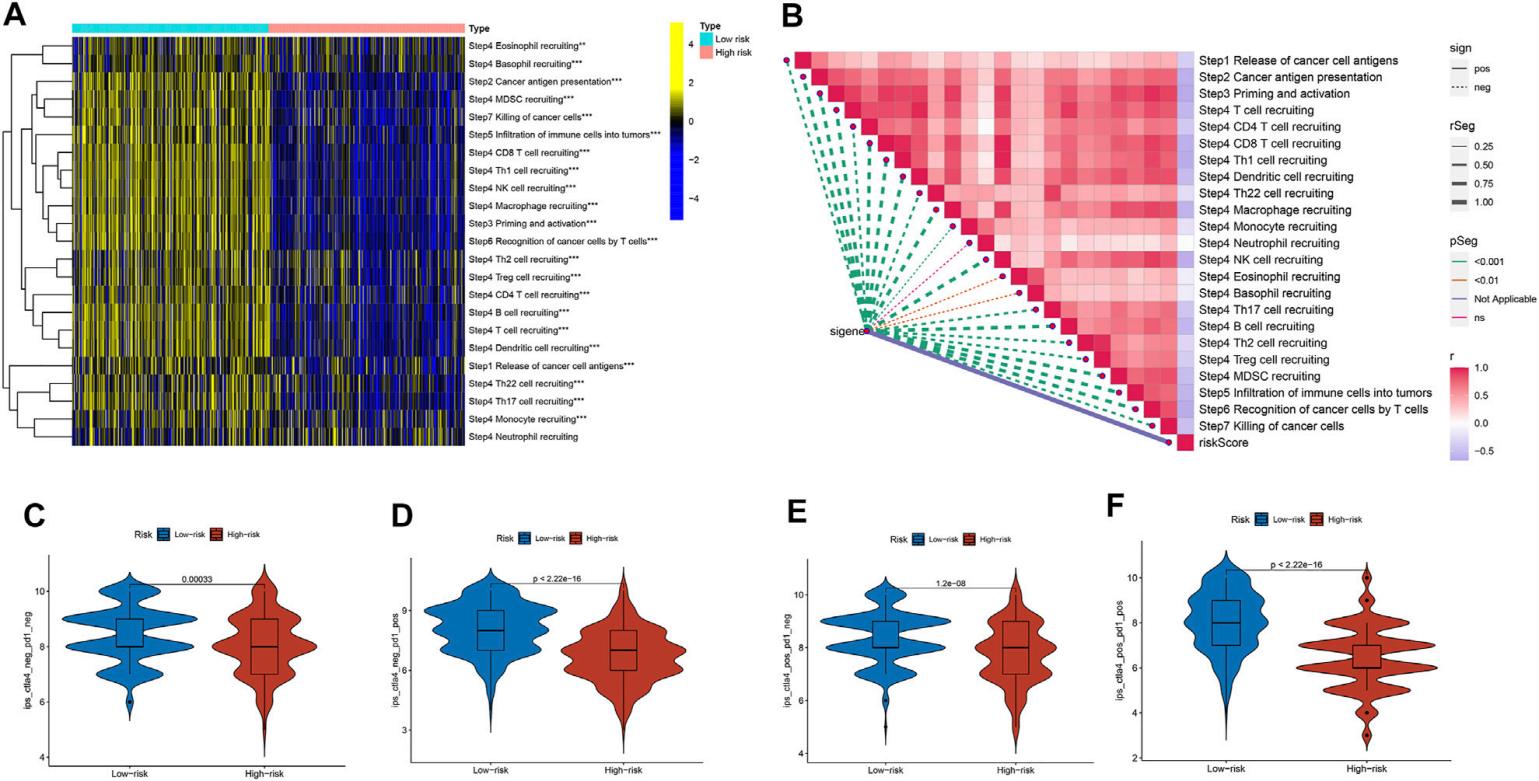
The cancer immunity cycle and the immunotherapy response prediction among different risk groups. **(A)** The enrichment of the cancer immunity cycle between the two risk groups. **(B)** The cancer immunity cycle and the risk score correlation. **(C,D,E,F)** The IPS and risk score correlation.

We explored the relationship between the risk score and the infiltrated immune cells’ abundance using the CIBERSORT-ABS, TIMER, XCELL, CIBERSORT, MCP-COUNTER, QUANTISEQ, and EPIC algorithms (Figure 7A). The ssGSEA algorithm had also been used to assess the immune cell infiltration levels associated with the risk score for 471 SKCM patients in the TCGA using transcriptome profiling data. Almost all cell types, related pathways, and functions were much higher in the low-risk group, except mast cell scores (*p* <0.05, Figures 7B,D). The gene expression and immune cells infiltration correlation were calculated using the CIBERSORT algorithm (Supplementary Figure S2). Immune infiltration can divide into four subtypes, named C1, C2, C3, and C4, which represent (wound healing), (INF-g dominant), (inflammatory), and (lymphocyte depleted), respectively, which have been used to demonstrate the correlation between the risk score and immune subtypes (Tamborero et al., 2018). The risk score was found to decline in C2 (Figure 7F) significantly. We assessed the relationships between the hub genes and the immune subtypes, FASLG, GATA3, TLR3, TNFRSF1B, and ZBP1, expressed at significantly higher levels in the C2 immune subtype (Supplementary Figures S3A–E), which was also significantly linked to TNFRSF21 downregulated gene expression (Supplementary Figure S3G). The TARDBP and PLK1 expression was significantly decreased in C3 (Supplementary Figure S3H). The TME and tumor stemness were important for tumor progression. To generate the immune, stromal, and ESTIMATE scores, the ESTIMATE algorithm had been used. The three scores differed significantly in the two risk groups (*p* <0.05; Figure 7G). The risk score and DNA and RNA methylation profiles had a positive correlation, which could measure the tumor stemness (DNAss, RNAss; *p* < 0.05; Figures 7C,E). The necroptosis-related hub gene expression, except USP22, PLK1, and TARDBP, correlated positively with stromal and immune scores (Supplementary Figures S4A–T). The cancer cells can escape anti-tumor immunity using an immunosuppressive mechanism of immune checkpoints. With the ICI therapy approved, ICIs have considerably transformed the clinical treatment of human cancer (Llovet et al., 2018; Salik et al., 2020). We then analyzed the relationship between the risk score and the immune checkpoint expression. The risk score and those immune checkpoint genes expression had a negative correlation, except the CD276 and VTCN1, which had a positive correlation with the risk score (Figure 7H). The results suggested that ICIs therapy was more suitable for the low-risk group. The cancer immunity cycle, which explains tumor cell immune detection and immunotherapy, has recently become a research hotspot. The cancer immunity cycle is divided into seven steps, from the initial antigen presentation until the final killing of tumor cells. As expected, all cancer immunity cycles were highly enriched in the low-risk group. The risk score and cancer immunity cycles had a negative correlation (Figures 8A,B).

### Analysis of Immunotherapy in the Risk Subgroups

Cancer immunotherapies, such as anti-CTLA4 and anti-PD1 therapies, improved the prognosis and OS in metastatic and advanced melanoma (Ladányi, 2015; Davis et al., 2019). Based on the background, we investigated the differences in the potential immunotherapeutic response between the two risk groups in the TCIA database. The prediction findings found that the more suitable for immunotherapy was the low-risk group (Figures 8C–F).

### Online Databases Verification

**FIGURE 9:**
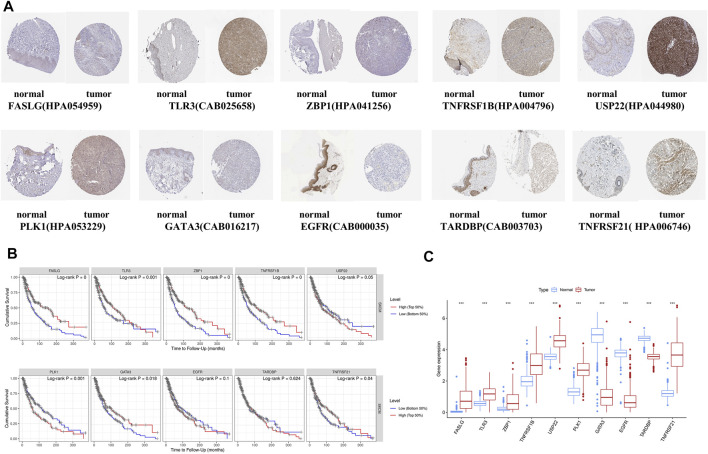
Online database analysis. **(A)** Validation of the 10-gene expression in our model based on the HPA database. **(B)** K-M curves for high- and low-expression level subgroups based on the 10-gene signature. **(C)** Hub gene expression in GTEx normal, TCGA normal, and TCGA cancer tissues.

To enhance the reliability of the database, the protein expressions of 10 NRGs were analyzed using the HPA database (Figure 9A). These results go along with our differential gene expression analysis (Figure 9C). The ten gene signatures’ Kaplan–Meier survival curves were presented in Figure 9B, and we found that USP22, PLK1, and EGFR high expression was significantly linked to poor prognosis.

### Correlation Analysis of Necroptosis-Related Genes and Tumor Microenvironment

**FIGURE 10:**
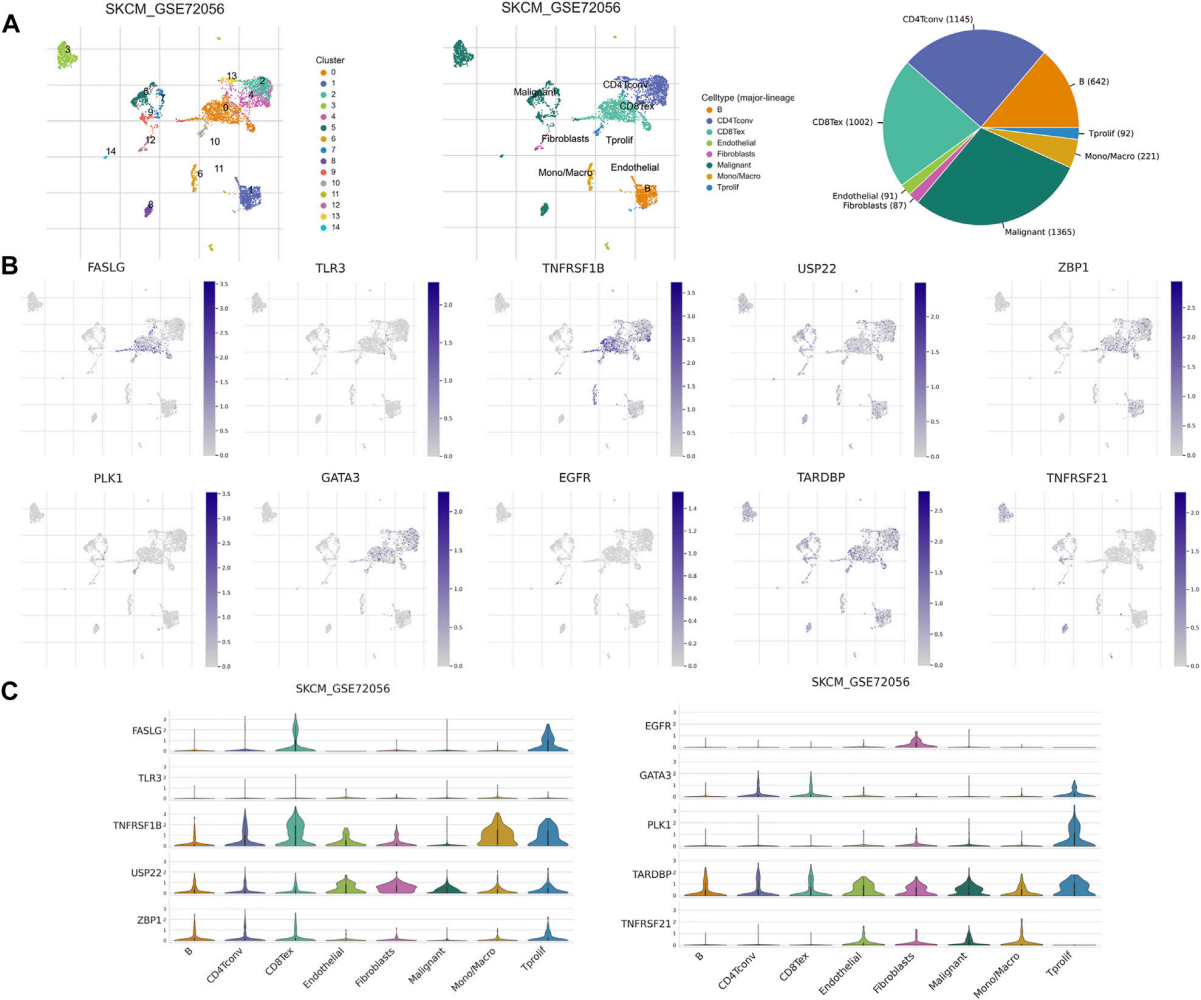
NRG expression in SKCM TME-associated cells. **(A)** Annotation of all cell types in GSE72056 and the percentage of each cell type. **(B,C)** Percentages and expressions of FASLG, TLR3, TNFRSF1B, USP22, ZBP1, PLK1, GATA3, EGFR, TARDBP, and TNFRSF21 in different cell types in GSE72056.

TME has an integral role in tumor occurrence, development, and prognosis. Therefore, we used six single-cell datasets (SKCM_GSE115978_aPD1, SKCM_GSE120575_aPD1aCTLA4, SKCM_GSE123139, SKCM_GSE139249, SKCM_GSE148190, and SKCM_GSE72056) from the TISCH database to analyze the expression of 10 NRGs in TME-related cells. We found that FASLG, TNFRSF1B, GATA3, ZBP1, and TARDBP had a high expression in a variety of immune cells, such as proliferating T cells, exhausted CD8^+^ T cells, CD4^+^ T cells, B cells, and NK cells. The highest expression of FASLG, TNFRSF1B, GATA3, TARDBP, and PLK1 was found in proliferating T cells, while ZBP1 showed the highest expression in plasma cells. In addition, TARDBP was also highly expressed in malignant cells and endothelial cells. USP22 is highly expressed in fibroblasts, endothelial cells, and malignant cells, and also has a low to moderate expression in immune cells of different types. EGFR was mainly expressed in fibroblasts, and TNFRSF21 was highly expressed in malignant cells and dendritic cells (Figures 10B,C, and Supplementary Figure S5). In GSE72056, there are 14 cell clusters and 8 cell types, and the distribution and number of various cell types have been visualized (Figure 10A).

## Discussion

Necroptosis, which is considered to be a secondary mechanism to apoptosis, is a tightly regulated inflammatory cell death form (Molnár et al., 2019). Necroptosis has been implicated in tumor initiation, progression, and metastasis, as indicated in previous research (Jouan-Lanhouet et al., 2014; Barbosa et al., 2018). Furthermore, necroptosis has been considered a novel approach to killing cancer cells and be a future treatment for cancer patients (Philipp et al., 2016). However, in previous studies, the NRG's specific role in the SKCM prognosis has not been fully elucidated.

In the current research, depending on NRGs from TCGA and GEO, a novel predictive model for SKCM was built and validated. We systematically investigated 67 NRGs in SKCM patients. The differentially expressed genes were screened, and after univariate Cox analysis and LASSO regression, we selected 10 genes (FASLG, TLR3, ZBP1, TNFRSF1B, USP22, PLK1, GATA3, EGFR, TARDBP, and TNFRSF21) to construct a novel 10-gene prognostic model. According to our assessment, this model predicted SKCM patients’ prognosis. The results and the identified genes are related to each other. For example, FASLG can reduce the melanoma cells’ mediated apoptosis to affect SKCM patients’ prognosis (Shukuwa et al., 2002). ADAR1 can suppress the ZBP1-mediated necroptosis to promote tumorigenesis (Karki et al., 2021). TLR3 directly activates necroptosis under the regulation of RIPK3 (Kaiser et al., 2013). As we know, this research is the first to present a new necroptosis-related prognostic model for predicting SKCM prognosis.

Before us, most literature concerning the prognostic gene signatures of SKCM was focused on m1A-, m5C-, and m6A-methylation (Wu et al., 2021); autophagy (Deng et al., 2021); ferroptosis (Xu et al., 2021); pyroptosis (Ju et al., 2021); and oxidative stress (Yang et al., 2021). For example, Yang et al. built an oxidative stress–associated gene’s prognostic model for melanoma. Deng et al. built the autophagy-related gene’s prognostic model for melanoma, which could predict the prognosis of SKCM efficiently. By contrast, we not only created a risk model but also comprehensively explored the link between the risk score and the immune response. Furthermore, our results were verified by the HPA database.

The development, prognosis, and treatment efficacy of melanoma were closely related to the TME (Avagliano et al., 2020). GSVA analyses revealed enriched pathways in the low-risk group, such as apoptosis, T-cell receptor signaling pathway, natural killer cell–mediated cytotoxicity, and toll-like receptor signaling. Many of these pathways were linked to necroptosis, cancer progression, and immunotherapy (Tang et al., 2020; Nouri et al., 2021). The GO analysis results showed the significant enrichment of genes in immune-related processes, such as immune response–activating signal transduction and lymphocyte-mediated immunity. The study findings indicated that the prognostic signature was correlated with melanoma tumor prognosis and related to the immune status of these cancer patients. We performed immune infiltration analysis by eight algorithms and found higher immune cell infiltration levels provoked higher immune pathway activation in the low-risk subgroup. This meant a decrease in the anti-tumor immunotherapy in the high-risk group.

Using ESTIMATE analysis, the low-risk group was found to have higher degrees of immune and stromal cell infiltration, which showed relatively good immunogenicity and immunoreactivity. By investigating immune infiltration, C2 was related closely to the low-risk score and might have the effect of preventing and inhibiting cancer progression. Furthermore, our results showed that cancer stem–like cell accumulation was positively correlated with a risk score. In recent decades, cancer stem–like cells are hypothesized to be responsible for cancer recurrence, therapy resistance, and metastasis. The cancer stem–like cells’ increased number and poor prognosis are correlated (Shiroki et al., 2017). Our research found a positive correlation between the risk signature and tumor stem cell scores, suggesting that our gene signature functions as a risk profile. The cancer immunity cycle is considered to be an important cyclic event for effective anti-tumor growth through immunity, and the cancer immunity cycle comprehensively reflects the outcome of a complex variety of immune regulatory interactions within the TME. A negative relationship between the risk score and the cancer immunity cycle step was found, and thus, the low-risk group was defined as an inflammatory TME. The immune checkpoints’ expression has a role in immune escape *via* inhibiting the T-cell response, and immune checkpoint inhibitors have been widely used for melanoma, especially anti-CTLA4 and anti-PD-1 antibodies (Carlino et al., 2021). Another feature of the inflammatory TME is the upregulation of immune checkpoint expression. In this research, the immune checkpoint genes’ expression and the low-risk group had a negative correlation. In addition, the two risk subgroups’ immunogenicity was evaluated using IPS analysis. These results meant the risk signature could guide the use of ICBs and the low-risk groups are suitable for immunotherapy. In addition to the existing therapies, the development of novel immunotherapeutic approaches holds great promise in the field of melanoma treatment. For example, the mutant P53 protein has been considered a new target for immunotherapy in melanoma, and the new biological drug ALT-801, which specifically targets P53 protein, is currently in a phase II clinical trial in combination with cisplatin in metastatic melanoma (Chasov et al., 2021).

TME consists of malignant cells, stromal cells, and immune cells, which play a key role in both tumorigenesis and metastasis (Arneth, 2019). FASLG, TNFRSF1B, GATA3, TARDBP, ZBP1, TNFRSF21, and TLR3 are mainly expressed in multiple immune cell types, and immune cells have a role in TME to inhibit tumor progression (Simiczyjew et al., 2020). We, therefore, speculated that the high expression of the above NRGs predicted a higher degree of immune cell infiltration in the TME, predicting a better prognosis. Subsequently, we compared the K-M curves of the two groups with high and low expression of NRGs in the TCGA cohort, and the groups with high expression of FASLG, TNFRSF1B, GATA3, TLR3, ZBP1, and TNFRSF21 had better survival times, thus well validating our hypothesis. USP22 mainly infiltrates endothelial cells. Since stromal cells can promote tumor growth and influence cancer behavior (Simiczyjew et al., 2020), we speculated that high expression of USP22 and EGFR suggested a higher degree of stromal cell infiltration in the TME, indicating a worse prognosis, and the K-M curve verified our hypothesis. PLK1 has been shown to be an oncogene (Li et al., 2018), and high PLK1 expression tends to be associated with reduced immune activity, which may be related to the fact that PLK1 is rarely expressed in other immune cells except for proliferating T cells. The K-M curves of the two groups with high and low expression of TARDBP were not statistically significant, and this may relate to the high expression of TARDBP in immune cells, malignant cells, and stromal cells. Therefore, these indicate that NRGs are associated with the TME of SKCM, and targeting the corresponding genes in corresponding cell types may benefit from manipulating the cellular components in the TME, but the specific mechanism needs further study.

Our study had some limitations. First, the research still requires a wider range of multi-center and prospective clinical research studies to support our hypothesis. Second, the present study consisted of only bioinformatic analyses, lacking verification through experiments *in vivo* and *in vitro.* Moreover, the detailed mechanism between NRGs and melanoma prognosis needs further investigation.

## Conclusion

We constructed a novel NRG risk signature in SKCM by combining bioinformatic tools and related algorithms. The ten-gene signature was linked to immune cell infiltration, TME, immune checkpoints, immune functions, and immunotherapy for SKCM patients. The results obtained from this study may contribute to the personalized clinical decision-making for SKCM patients.

## Data Availability

The datasets presented in this study can be found in online repositories. The names of the repository/repositories and accession number(s) can be found in the article/Supplementary Material.
