# Supplementary material for: A Novel Necroptosis-Related Gene Signature in Skin Cutaneous Melanoma Prognosis and Tumor Microenvironment
Source: Front Genet. 2022 Jul 11;13:917007. doi: 10.3389/fgene.2022.917007 (PMC9309482; doi:10.3389/fgene.2022.917007)
Supplement: Supplementary file 4 [file DataSheet1.docx]

Supplementary Material

# Supplementary Figures

## Supplementary Figures
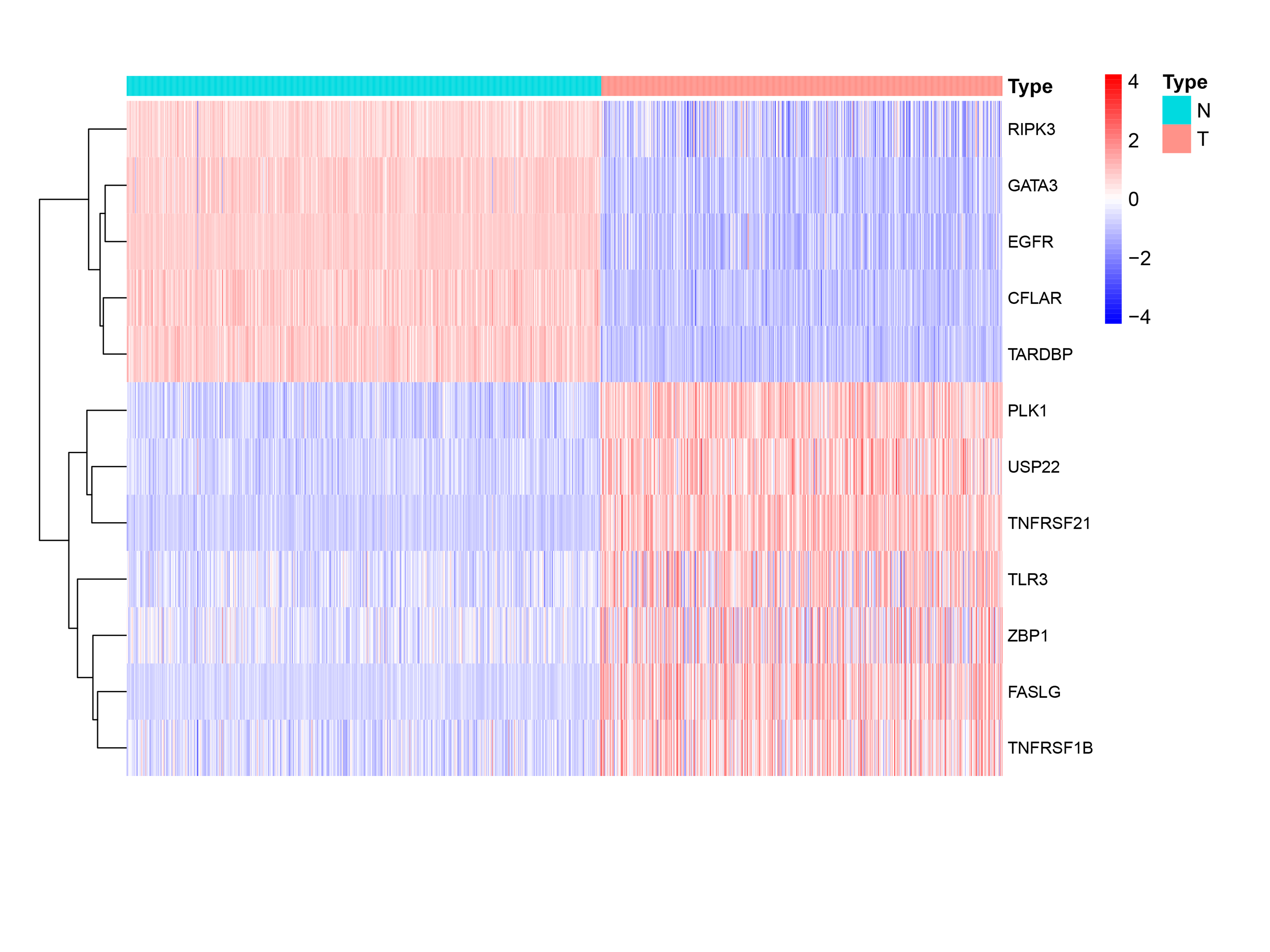
[Supplementary](javascript:;) Figure S1. The prognostic DEGs between tumors and normal tissues


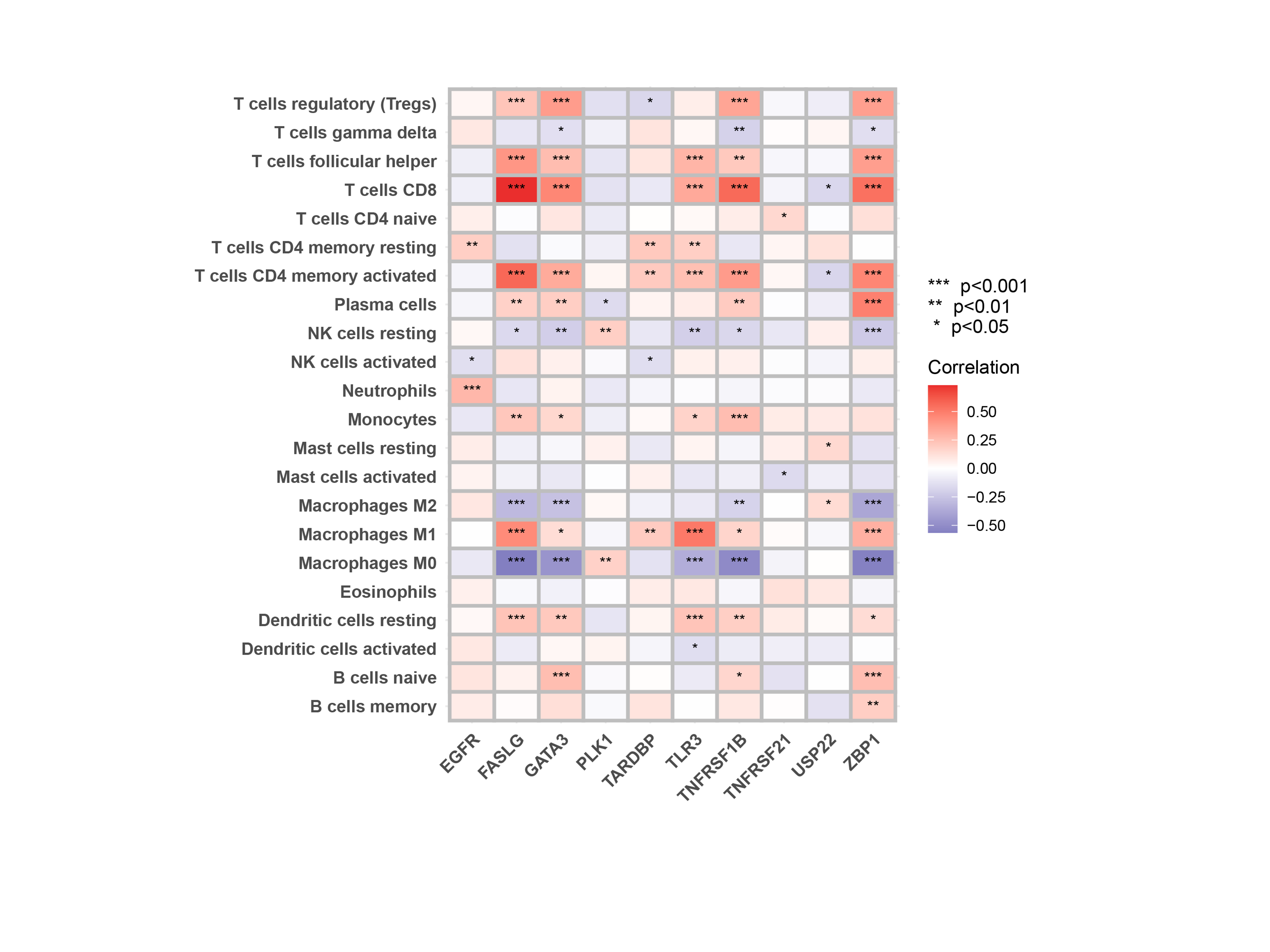


[**Supplementary**](javascript:;) **Figure S2.** The hub genes expression and immune cells infiltration’s relationship by CIBERSORT algorithm.

**
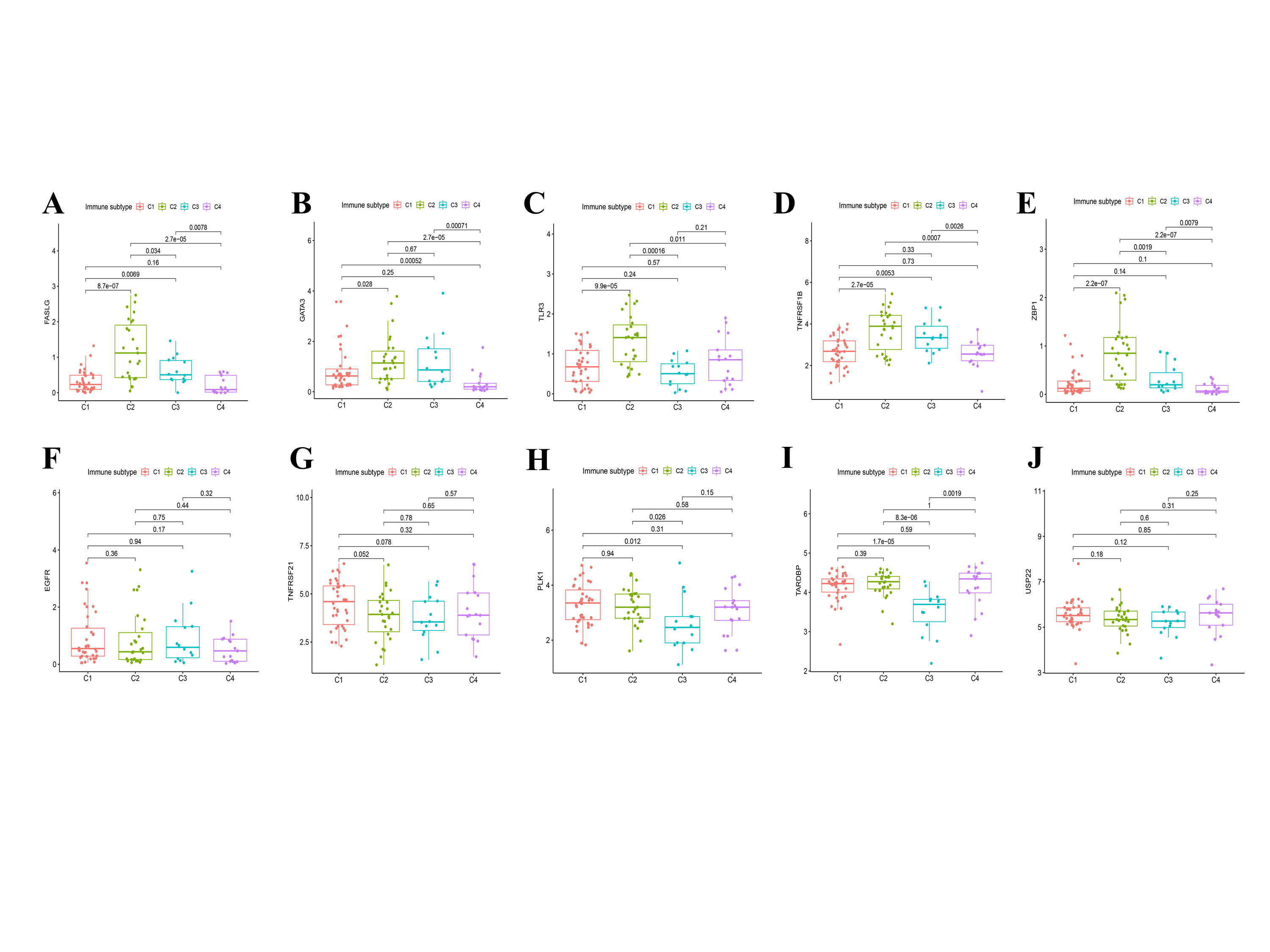
**

[**Supplementary**](javascript:;) **Figure S3.** Expression of NRGs including FASLG (**A**), GATA3 (**B**), TLR3 (**C**), TNFRSF1B (**D**), ZBP1 (**E**), EGFR (**F**), TNFRSF21 (**G**), PLK1 (**H**), TARDBP (**I**), and USP22 (**J**) in four subtypes of immune infiltration.

**
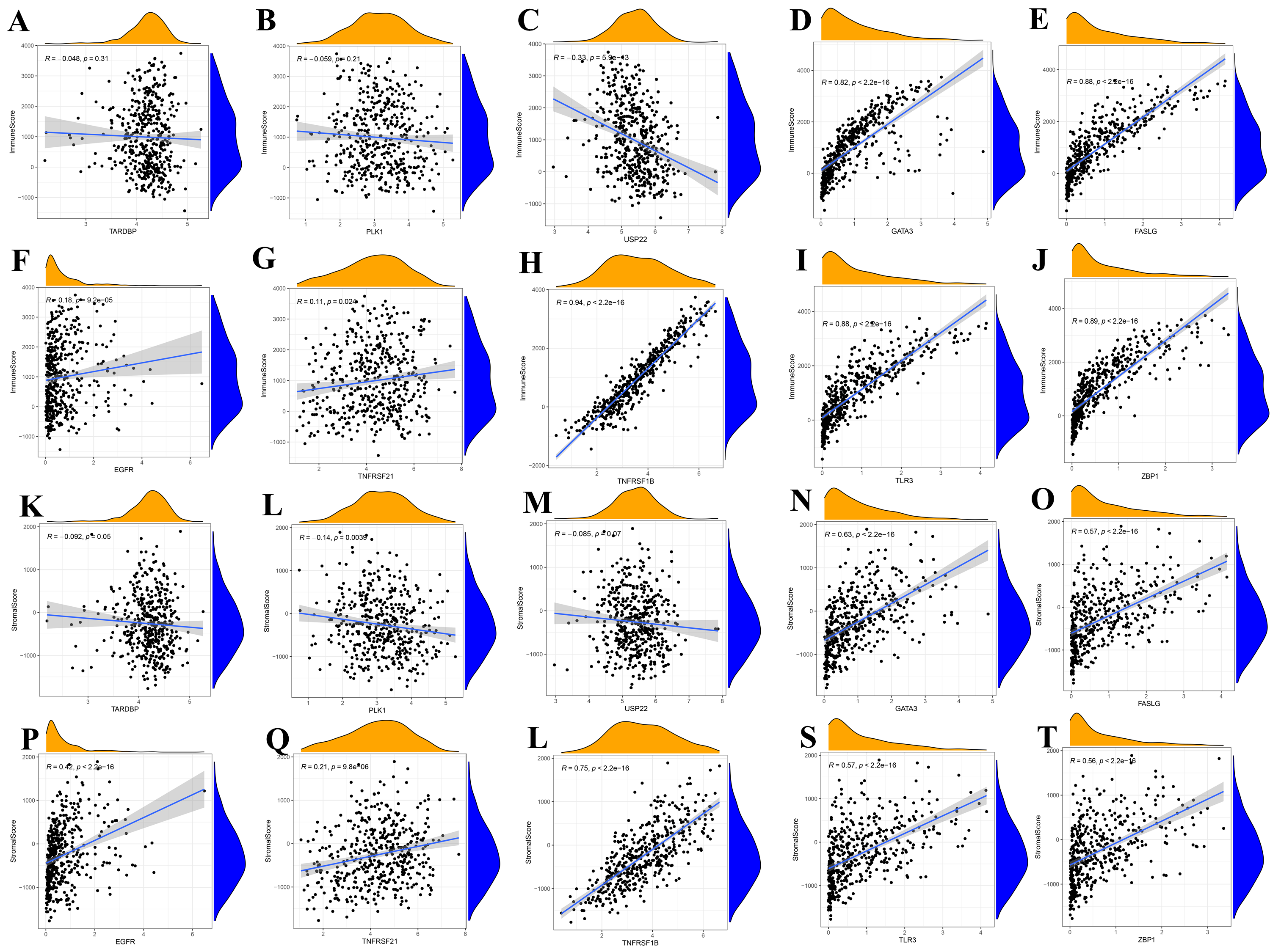
**

[**Supplementary**](javascript:;) **Figure S4.** Association between hub genes and immune and stromal scores, including TARDBP (**A, K**), PLK1 (**B, L**), EGFR (**C, M**), FASLG (**D, N**), GATA3 (**E, O**), TLR3 (**F, P**), TNFRSF1B (**G, Q**), TNFRSF21 (**H, L**), USP22 (**I, S**), and ZBP1 (**J, T**).


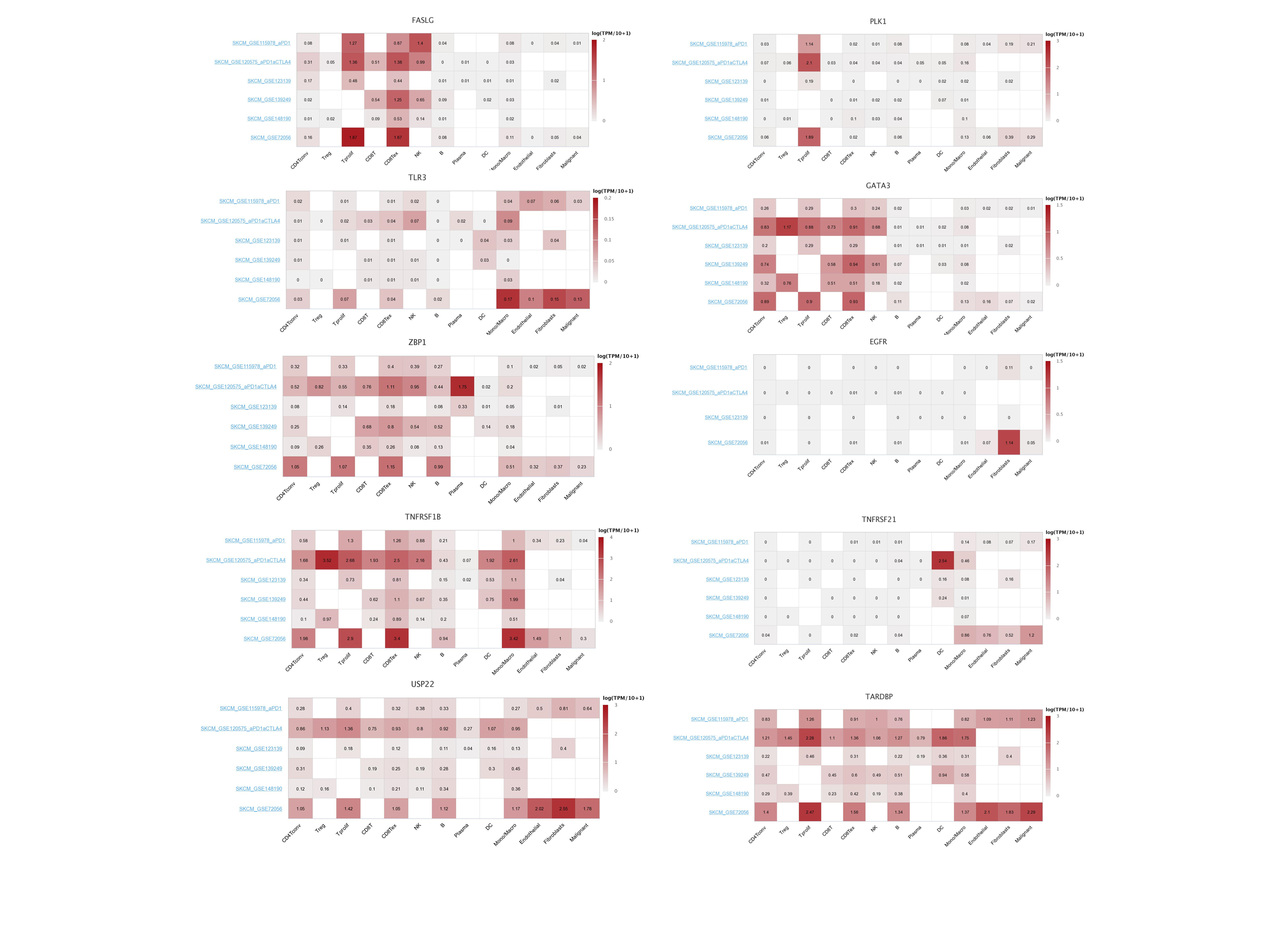


[**Supplementary**](javascript:;) **Figure S5.** Expression levels of NRGs in SKCM TME-associated cells in the GEO database
